# Supplementary material for: Successional Shifts in Soil C:N:P stoichiometry and its environmental controls during forest restoration in Huize County, Qujing, Yunnan Province
Source: PLoS One. 2026 Aug 21;21(8):e0356261. doi: 10.1371/journal.pone.0356261 (PMC13496410; doi:10.1371/journal.pone.0356261)
Supplement: Supplementary file 2 [file pone.0356261.s004.zip › Data and Code/RF/mixed conifer–broadleaf forest/figures/importance_C.N.pdf]

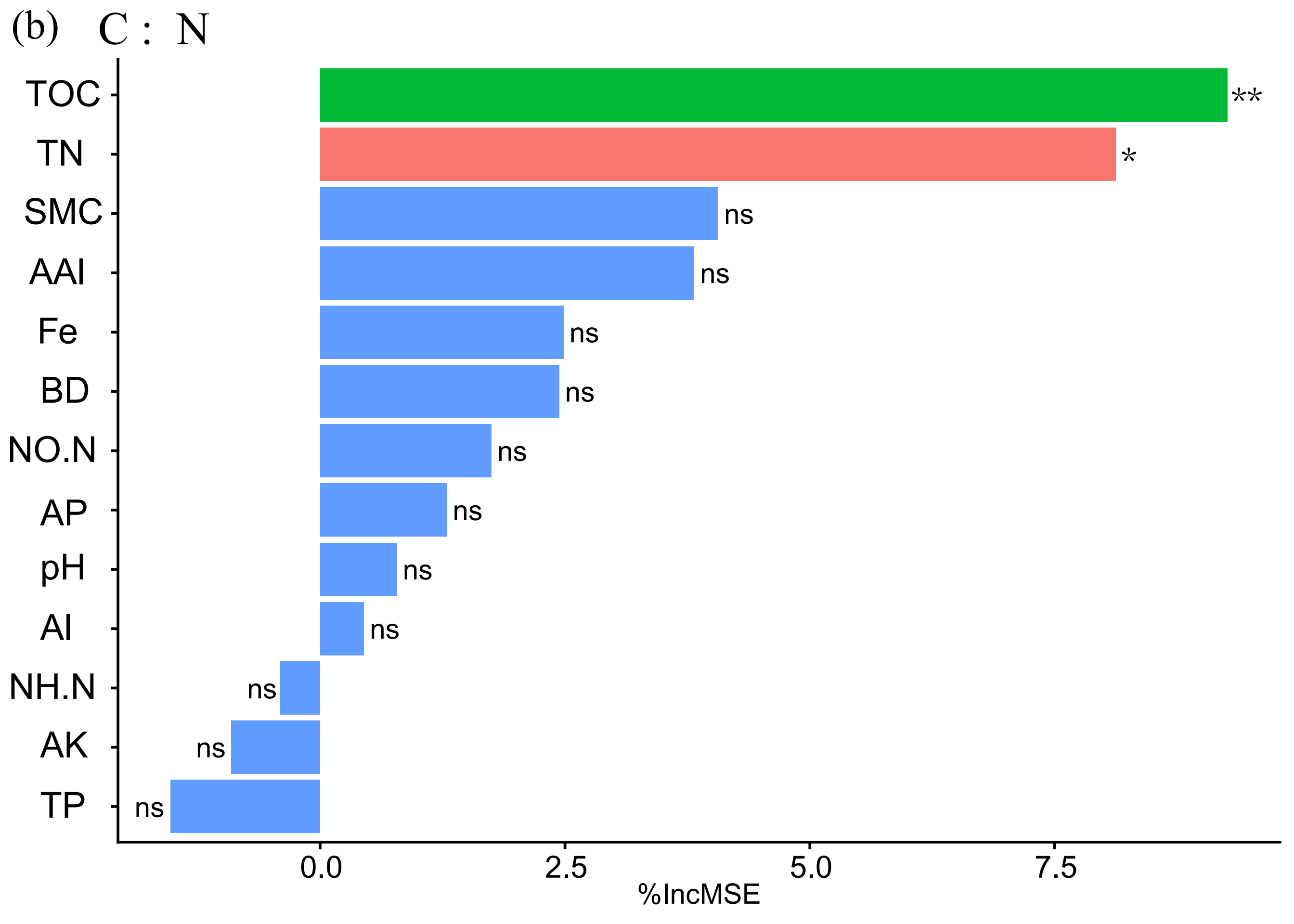

(b) C : N
TOC
TN
SMC
AAI
Fe
BD
NO.N
AP
pH
Al
NH.N
AK
TP
**
*
ns
0.0
2.5
5.0
7.5
%IncMSE
